# Baseline income, follow-up income, income mobility and their roles in mental disorders: a longitudinal intra-generational community-based study

**DOI:** 10.1186/s12888-020-02578-0

**Published:** 2020-04-22

**Authors:** Xiangfei Meng, Aihua Liu, Carl D’Arcy, Jean Caron

**Affiliations:** 1 Department of Psychiatry, McGill University, Montreal, Canada; 2 Douglas Research Centre, Montreal, Canada; 3 Department of Psychiatry, University of Saskatchewan, Saskatoon, Canada; 4 School of Public Health, University of Saskatchewan, Saskatoon, Canada

**Keywords:** Social causation, Income, Depression, Mental disorder

## Abstract

**Background:**

Although a number of studies have found that income mobility associated with an elevated risk of mental disorders, existing research does not provide sufficient evidence of how exactly individuals’ experience of income mobility per se affects their risk of mental health outcomes. This present study aimed to explore roles of baseline income, follow-up income, and income mobility in the development of mental disorders using an intra-generational, longitudinal follow-up study.

**Methods:**

We used data from the Montreal South-West Longitudinal Catchment Area Study. A total of 1117 participants with complete information both on income and past 12-month diagnoses of mental disorders were selected for this study. Diagonal Reference Models were used to simultaneously examine roles of income at baseline, income at follow-up, and income mobility in mental disorders during a 4-year follow-up.

**Results:**

Both baseline and follow-up income were important predictors for any mental disorder and major depression among males and females. Those with low income had a higher risk of any mental disorders and major depression. No evidence was found to support an association between income mobility (neither downwards nor upwards) and mental disorders. Marital status was uniquely associated with any mental disorder among males. Having a pre-existing diagnosis of any mental disorder at origin was associated with any mental disorder and major depression at the end of the 4-year follow-up.

**Conclusions:**

This study first simultaneously examined roles of income at baseline, at follow-up, and mobility in mental disorders among a large-scale intra-generational community-based study. This present study provides additional evidence on how income is associated with an individuals’ likelihood of mental disorders.

## Background

Income profoundly influences health and human capacity [1]. Low income increases the risk of developing a wide range of health-related determinants, such as poor nutrition, poor housing, and less access to necessary health services [2–4]. These stressful events and precarious pre-existing issues (e.g. poor housing, financial, employment, and relationship conditions) could then trigger mental disorders [5]. This impact could be even passed by to the next generation [6]. There are two major hypotheses (health selection and social causation) proposed to explain the causal direction of the socioeconomic status – health relationship [7], but there is no consistent evidence to support one hypothesis over another [8].

Income mobility is a change in income relative to one’s original income location within a given society. A high degree of income mobility, either upwards or downwards, indicates the stratification system of a society is open. The relationship between income mobility and health has profound ethical and political implications in the pernicious effects of human inequality and the differential impact on social classes of economic and social policies [9, 10].

Although a number of studies have found that income mobility associated with an elevated risk of mental disorders [11–18], existing research does not provide sufficient evidence of how exactly individuals’ experience of income mobility per se affects their risk of mental health outcomes. Investigating this question requires one to disentangle the separate effects of income at baseline, income at follow-up, and income mobility simultaneously. The literature to date has mainly been based on conventional methods, which can only estimate social mobility, social origin, and social destination, individually. These conventional approaches do not consider complex relationships between social origin, destination, and mobility.

One analytical approach that allows for simultaneous estimation of effects is the Diagonal Reference Models (DRMs). DRMs can estimate all three factors simultaneously. Recently more studies have begun to apply this approach to explore the effects of social mobility on health outcomes [19, 20]. A recent study on the effects of education mobility on overweight and obesity compared conventional approaches and DRMs in their analyses and found that the results from DRMs modeling to be superior [21]. They recommended the use of DRMs in evaluating the consequences of all different types of social mobility on health. Though DRMs are being used more in research on a broad range of social phenomena, few studies have been carried out to apply this approach in longitudinal data to disentangle the mobility effect from the origin and destination effects in a longitudinal data [22].

We believe that investigating the role of intra-generational income mobility could help us understand the social determinant of mental disorders. The present study aimed to explore roles of income at baseline and income at follow-up in major depression "and any mental disorder" and to examine how income mobility influenced mental disorders by applying DRMs methodology in an intra-generational, longitudinal follow-up study. We also investigated factors that were involved in the intra-generational transmission of income mobility on mental disorders.

## Methods

### Data source

The Montreal South-West Longitudinal Catchment Area Study—Zone d’Épidémiologie Psychiatrique du Sud-Ouest de Montréal (ZEPSOM), is a longitudinal population-based cohort study of a representative community sample of five neighbourhoods in the South-West sector of Montreal, Canada, which had a target population of 269,720.

The interviewers conducted a scheduled face-to-face meeting either at participants’ homes or in a research facility. To ensure the quality and consistency of data collected, all the interviewers have a university education in humanities; they received 5 days of training and were supervised every week. Data were collected using laptop computers, which transferred the information directly into the database of the study. The interview generally ranged from 1.5 to 3 hrs, depending on whether a mental disorder was discovered. The interview included a wide range of validated questionnaires on sociodemographic factors, psychological distress, and a set of common mental and behavioral problems. More detailed information about this study could be found in previously published articles [23–25].

### Study sample

To examine the roles of baseline income, follow-up income, and income mobility in mental disorders, this current study only included participants who conducted the data collections at the baseline (2007/8) and the follow-up (2012/3). A total of 1117 participants with complete information both on income and past 12-month diagnoses of mental disorders were selected for this study.

### Measures

#### Outcome

Past 12-month mental disorders were identified by the Canadian Community Health Survey (CCHS) version 1.2 of the Composite International Diagnostic Interview (CIDI), including major depression, mania, panic disorder, social phobia, agoraphobia, and alcohol and drug dependence [26, 27]. We used “any mental disorder” to define the presence of any kind of studied mental disorders in the past 12-month prior to the interview. Because of the low incidence of most selected mental disorders assessed in the survey, major depression was the only specific mental disorder that could be analyzed separately.

#### Independent variable

Income was categorized into two categories low-income and non-low income. The low-income measures developed by Statistics Canada were used to identify the low-income population. Individuals with an adjusted family income that was lower than 50% of the median Canadian level were classified as low income. We used this binary variable to indicate income in the societal context and reflect economic fluctuations over the 4-year follow-up period in the study area.

#### Covariates

In addition to the effects of baseline and follow-up incomes, we also controlled for a variety of variables (*age, sex, immigrant status, marital status, level of education, whether or not having a family history of mental health conditions, and whether or not the study participant had a lifetime diagnosis of any mental disorder at baseline)* in our DRMs models. The Parental Psychiatric History [19] was used to assess if a biological parent had been treated by a psychiatrist or hospitalized for mental health problems, or experienced any of the following problems, depression, delirium or hallucination, anxiety disorders, substance abuse or suicide.

### Statistical analyses

To reflect sex differences in mental health and social context, we analyzed males and females separately. The DRMs were used to test the roles of income at baseline and follow-up positions and income mobility on mental health outcomes. DRMs were developed for the purpose of empirically disentangling mobility effects from baseline and follow-up effects, and have demonstrated their utility in estimating health consequences of social mobility [19–21]. DRMs can estimate the effects of mobility and positions of baseline and follow-up [20]. DRMs estimate mobility effects using the following equation:

$$ {Y}_{ijk}=w\times {\mu}_{ii}+\left(1-w\right)\times {\mu}_{jj}+\sum {\beta}_{ijk l}+{e}_{ijk} $$

where *Y*_*ijk*_ is the value of outcome in cell *ij* of the mobility table which has *k* observations, *μ*_*ii*_ is the estimated mean of Y in the diagonal cell in the row denoting the position at baseline, whereas *μ*_*jj*_ is the estimated mean of Y in the diagonal cell in the row denoting the position at follow-up, w estimates the contribution of baseline relative to follow-up, lies in the interval [0,1]. Using the w-parameter, the two effects are combined in an intercept for each cell. This method allows examining the effect of social mobility in addition to baseline and follow-up effects [28].

Our consideration of income mobility effects on mental health outcomes included baseline and follow-up effects of downwards or upwards mobility, and other relevant covariates. We used non-linear modeling in all the analyses. STATA version 15 subcommand DRM was used to analyze the DRM in this study [29].

## Results

### A summary of the study cohort

The mean age of the study cohort at follow-up was 43.9 (SD = 12.8) years. The cohort included more females (63.9, 95% CI 61.1–66.7%) than males (36.1, 95% CI 33.3–38.9%). A total of 200 participants (17.9, 95% CI 15.8–20.3%) had a lifetime diagnosis for any mental disorder at baseline. Overall, this study cohort had a reduced prevalence of low income over the 4-year follow-up. The proportion of low income at baseline was 19.8% (95% CI 17.5–22.2%) and this proportion dropped to 17.6% (95% CI 15.5–20.0%). The majority of participants, 87.6% (95% CI 85.6–89.5%) did not experience income mobility with 5.10% of the study sample (95% CI 3.95–6.56%) reporting downwards mobility and 7.25% (95% CI 5.87–8.93%) reporting upwards mobility.

### Effects of baseline income, follow-up income, and income mobility on any selected mental disorder and major depression

We ran diagonal reference models for outcomes of any mental disorder and major depression, including baseline income, follow-up income, income mobility and a number of covariates (age, immigrant status, whether or not being a Caucasian, marital status, level of education, whether or not having a family history of mental disorder, whether or not having a history of lifetime mental disorder at the origin) in the models. Table 1 shows the estimated odds from DRMs for any mental disorder. The odds of having any mental disorder in the past 12-month for income immobile males in the low-income category was 0.19, whereas the immobile males who were not in the low-income category had much lower odds of having any mental disorder (0.09). A lower level of income was associated with a higher likelihood of any mental disorder for both females and males. The weight of baseline income is an indicator of relative effects the origin position had on mobile men compared to the effects of destination. Its value ranges from 0 to 1. For most of our analyses, the weight of origin was 0 or constrained. As the parameter of 0 differs from 0.5 (null hypothesis = origin and destination has an equal contribution to outcomes), it may suggest that mobile males were differently influenced by their baseline income and follow-up income. This was also true for females. The model also found downwards income mobility had no additional effect on the odds of having any mental disorder (*p* = 0.528). Similarly, no upwards income mobility was identified for any mental disorder (*p* = 0.486). Marital status was significantly associated with the odds of having any mental disorder. Males who were married or separate, widowed or divorced had lower odds of having any mental disorder compared to those who were single.

**Table 1:** Effects of baseline income, follow-up income, and income mobility on any studied mental disorder among males and females from diagonal reference models (DRMs) with 95% confidence intervals

	Males			Females		
	Coefficients	95% CI	*P-value*	Coefficients	95% CI	*P-value*
**Model 1**						
Diagonal intercepts^a^						
Origin						
Low	0.19	0.09,0.29	< 0.001	0.17	0.11,0.23	< 0.001
Not low	0.09	0.03,0.15	0.003	0.06	0.03,0.08	< 0.001
Weight of origin	Constrained			0.001		
Downwards mobility	0.05	−0.11,0.21	0.528	0.02	−0.09,0.13	0.720
Marital status						
Single	Reference					
Married	−0.08	−0.16, − 0.01	0.028			
Common law	0.03	−0.06, 0.12	0.521			
Separate or widowed or divorced	−0.09	− 0.18,− 0.00	0.042			
Presence of any mental disorder at origin	0.23	0.15,0.31	< 0.001	0.08	0.02,0.14	0.011
**Model 2**						
Diagonal intercepts^a^						
Origin						
Low	0.17	0.08, 0.27	< 0.001	0.17	0.11–0.23	< 0.001
Not low	0.07	0.02–0.13	0.010	0.05	0.03,0.08	< 0.001
Weight of origin	−0.00			-0.00		
Upwards mobility	− 0.04	− 0.16,0.08	0.486	0.04	−0.04,0.12	0.316
Marital status						
Single	Reference					
Married	−0.06	−0.14,0.01	0.077			
Common law	0.04	−0.04,0.13	0.260			
Separate or widowed or divorced	−.06	−0.15,0.03	0.161			
Presence of any mental disorder at origin	0.23	0.15,0.31	< 0.001	0.09	0.04,0.14	0.002

^a^The diagonal intercepts are odds

For females, we found that low-income immobile females had higher odds of having any mental disorder in the past 12-month. There was no statistical difference in marital status. Again, mobile females might also be differently influenced by their income at baseline and follow-up. Having a prior diagnosis of any mental disorder was consistently linked with the subsequent occurrence of any mental disorder among males and females during the study period (*p* < 0.001). Neither downwards nor upwards income mobility was associated with the occurrence of any mental disorder (*p* > 0.05). Table 2 provides the income mobility trajectories of all the participants included in the analyses.

**Table 2:** Overview of intra-generational income mobility among males and females

Origin (Income at baseline)	Males Destination (Income at the 4-year follow-up)			Females Destination (Income at the 4-year follow-up)		
	*μ*_1_ Low	*μ*_2_ High	Total	*μ*_1_ Low	*μ*_2_ High	Total
*μ*_1_ Low	***μ***_**11**_ **43**	*μ*_12_ 24	67	***μ***_**11**_ **97**	*μ*_12_ 57	154
*μ*_2_ High	*μ*_21_ 19	***μ***_**22**_ **317**	336	*μ*_21_ 38	***μ***_**22**_ **522**	560
Total	62	341	403	135	579	714

Immobile individuals on diagonals (bold), and individuals with downwards mobility below diagonals, whereas individuals with upwards mobility above diagonals

For major depression (Table 3), low-income immobile males and females had higher odds of having major depression. Income mobile participants may be differently influenced by their income at baseline and follow-up. Having a prior diagnosis of any mental disorder at origin was consistently associated with a higher risk of major depression among males and females (*p* < 0.001). Neither downwards nor upwards income mobility among males and females was associated with major depression at follow-up (*p* > 0.05). Consistently, both income at baseline and follow-up played significant roles in major depression (*p* < 0.05). Having a lifetime diagnosis of any mental disorder at baseline predicted a greater risk of major depression at follow-up.

**Table 3:** Effects of baseline income, follow-up income, and income mobility on major depression among males and females from diagonal reference models (DRMs) with 95% confidence intervals

	Males			Females		
	Coefficients	95% CI	*P-value*	Coefficients	95% CI	*P-value*
**Model 1**						
Diagonal intercepts^a^						
Origin						
Low	0.04	−0.03,0.11	0.253	0.14	0.09,0.20	< 0.001
Not low	0.03	0.00,0.06	0.029	0.04	0.02,0.06	0.001
Weight of origin	Constrained				0.00	
Downwards mobility	0.05	−0.08,0.17	0.480	0.03	− 0.07,0.12	0.609
Presence of any mental disorder at origin	0.17	0.10,0.23	< 0.001	0.06	0.01,0.12	0.015
**Model 2**						
Diagonal intercepts^a^						
Origin						
Low	0.05	−0.02,0.12	0.175	0.15	0.10,0.20	< 0.001
Not low	0.03	0.01,0.06	0.013	0.04	0.02,0.06	< 0.001
Weight of origin	−0.00			0		
Upwards mobility	−0.05	− 0.15,0.04	0.274	− 0.04	− 0.11,0.03	0.239
Presence of any mental disorder at origin	0.15	0.09,0.21	< 0.001	0.05	0.01,0.10	0.024

^a^The diagonal intercepts are odds

We also tested the effects of baseline income, follow-up income, and income mobility on any mental disorder and major depression in the total study sample and found similar results (Table 4). No interactions among studied covariates were associated with any mental disorder or major depression (*p* > 0.05).

**Table 4:** Effects of baseline income, follow-up income, and income mobility on any mental disorder and major depression from diagonal reference models (DRMs) with 95% confidence intervals

	Any mental disorder			Major depression (*N* = 1053)		
	Coefficients	95% CI	*P-value*	Coefficients	95% CI	*P-value*
**Model 1**						
Diagonal intercepts^a^						
Origin						
Low	0.19	0.13,0.24	< 0.001	0.11	0.07,0.15	< 0.001
Not low	0.09	0.06,0.13	< 0.001	0.04	0.02,0.05	< 0.001
Weight of origin	−0.003			Constrained		
Downwards mobility	0.04	−0.05,0.13	0.403	0.03	−0.05,0.11	0.463
Marital status						
Single	Reference					
Married	−0.06	−0.11,− 0.02	0.010			
Common law	-0.02	−0.07,0.04	0.537			
Separate or widowed or divorced	−0.06	− 0.11,− 0.01	0.026			
Presence of any mental disorder at origin	0.12	0.08,0.17	< 0.001	0.10	0.06,0.14	< 0.001
**Model 2**						
Diagonal intercepts^a^						
Origin						
Low	0.17	0.12,0.23	< 0.001	0.11	0.07,0.16	< 0.001
Not low	0.08	0.04,0.12	< 0.001	0.04	0.02,0.05	< 0.001
Weight of origin	0.01			0.001		
Upwards mobility	0.01	-0.01,0.07	0.923	−0.05	−0.10,0.01	0.079
Marital status						
Single	Reference					
Married	−0.05	−0.09,− 0.01	0.044			
Common law	-0.01	−0.06,0.04	0.743			
Separate or widowed or divorced	−0.04	− 0.09,0.01	0.153			
Presence of any mental disorder at origin	0.13	0.09,0.18	< 0.001	0.09	0.05,0.12	< 0.001

^a^The diagonal intercepts are odds

## Discussion

This study first simultaneously examined the roles of baseline income, follow-up income, and income mobility in mental disorders among a large-scale intra-generational community-based study. We found that both baseline income and follow-up income were important predictors of any mental disorder and major depression among males and females. Those with low income had a higher risk of any mental disorders and major depression at follow-up. No evidence was found to support an association between income mobility (neither downwards nor upwards) and mental disorders. Marital status was only associated with any mental disorder among males. Having a pre-existing diagnosis of any mental disorder at baseline was linked with a higher risk of any mental disorder and major depression at the end of the 4-year follow-up.

As discussed in the Introduction, the social gradient (income) in mental disorders is well established [7, 8]. Participants in the low-income category were at increased odds of any mental disorders compared with those in the not low-income category. The main findings of this study have important public health implications and substantial policy implications. Most important, the findings suggest that low-income is associated with substantial psychopathology and that there is a need for targeted interventions to prevent and treat mental disorders in the population with low-income.

In terms of income mobility, studies have found a lagged effect of income on subsequent health outcomes [30]. It can take up to 6 years to observe the negative consequences of low income on health [31]. Likewise, we found that people with low income at baseline and the follow-up were more likely to report any mental disorder or major depressive episode at the end of the 4-year follow-up. One possible explanation for this is that low income had a long-lasting effect on mental disorders. This is in line with the social causation theory, which hypothesizes that low income leads to negative health outcomes [32]. In addition, social causation theory generally suggests the genetic liability to a disease interacts with poverty in developing disease [33]. Many studies have suggested the interplay between life stress and different genetic variants, such as serotonin transporter promoter variant [34–36]. Although we considered whether or not having a family history of mental disorders as a covariate, which might indicate the genetic susceptibility of mental disorders, no significant associations were identified between whether or not having a family history of the occurrence of mental disorders and any mental disorder or major depression at the end of the 4-year follow-up. Questions as to what extent genetic variations, and how these variations, interact with poverty in mental disorders remain to be answered.

Notably, our findings suggest that baseline income may have a different role in any mental disorder and major depression compared to follow-up income. As a result of most of our findings with 0 or constrained value for the weight of baseline income for the outcomes, further research is warranted to scrutinize the relative importance of baseline income versus follow-up income.

We did not find income mobility (neither upwards nor downwards) was associated with the occurrence of any mental disorder or major depression at the end of the 4-year follow-up. There are several possible explanations for this. First, changes in income positions might take a longer time to manifest the effect of income-induced stress attributed to any mental disorder and major depression. This income-induced stress could include upwards (people having high-income later-on and suffering from the societal stress of not familiar with their peers’ social norms) and downwards (people making less income later-on and struggling to provide basic living demands and dealing with the stress to meeting the basic needs). Second, the role of income mobility in mental disorders may be mediated by other covariates. It may largely be involved in externalizing disorders. Costello et al. [37] used a natural experimental study to examine the relationship between poverty and psychopathology among children and adolescents. They found that moving families out of poverty had a major effect in lowering behaviour problems, particularly conduct and oppositional disorders. Parental supervision was a significant mediator for the income mobility-conduct disorders relationship. When people moved out of poverty, parents tended to spend more time with their children, which in turn reduced the risk of externalizing disorders. Third, only substantial changes in income positions may relate to health outcomes. A Swedish study found only an increase in income over a 5-year period was associated with improvement in self-rated health [31]. Our current study only captured income data on two categories.

In line with the literature [38], we found that a pre-existing diagnosis of mental disorder was associated with a higher risk of any mental disorder and major depression at follow-up. We did not find having a positive family history of mental disorders associated with any mental disorder or major depression. Having a family history of mental health conditions, such as schizophrenia, major depression, has been associated with a higher risk of mental disorders among offspring [39]. One possible explanation for the finding in our study is that the competing risk between whether or not having a family history of mental disorders and whether or not having a pre-existing diagnosis of mental disorders at baseline in the final model. Compared to the family history of mental disorders, having a pre-existing diagnosis of mental disorders was a stronger risk predictor for developing a subsequent mental disorder.

### Strengths and limitations

One of the apparent strengths of the present study is that it simultaneously examined the roles of baseline income, follow-up income, and income mobility in mental disorders. Also, this study demonstrated the possibility of applying DRMs to longitudinal data on mental health outcomes.

There are several limitations to be noted. First, we were limited to examine the relationships between income mobility and all studied mental disorders, except major depression, due to the small number of people developing other mental disorders. Second, income was dichotomized into low and not low groups after adjustment for household size. The data originally collected in the past did not allow us to further specify income categories. Third, the data analyzed in this study covered 4 years. The role of income mobility in mental disorders may take longer to manifest. Finally, we cannot disentangle the underlying mechanisms between roles of baseline income, follow-up income, and income mobility and mental disorders. We cannot infer causality in the relationship between income and mental disorders.

## Conclusions

This present study provides additional evidence on how income is associated with an individuals’ likelihood of mental disorders. Males and females with low-income at baseline were more likely to develop mental disorders at follow-up. During the study period, neither upwards nor downwards income mobility was associated with developing a mental disorder.

## Data Availability

This study uses a dataset from an ongoing prospective cohort study. Permission was granted for analyzing the current dataset. Interested researchers could contact authors for the access.

## Abbreviations

DRMs: Diagonal Reference Models
ZEPSOM: Zone d’Épidémiologie Psychiatrique du Sud-Ouest de Montréal
CCHS: Canadian Community Health Survey
CIDI: Composite International Diagnostic Interview
SD: Standard Deviation
CI: Confidence intervals
